# Metformin Alleviates LPS-Induced Acute Lung Injury by Regulating the SIRT1/NF-κB/NLRP3 Pathway and Inhibiting Endothelial Cell Pyroptosis

**DOI:** 10.3389/fphar.2022.801337

**Published:** 2022-07-15

**Authors:** Yunqian Zhang, Hui Zhang, Siyuan Li, Kai Huang, Lai Jiang, Yan Wang

**Affiliations:** Department of Anesthesiology and Surgical Intensive Care Unit, Xinhua Hospital Affiliated to Shanghai Jiaotong University School of Medicine, Shanghai, China

**Keywords:** ARDS, endothelial pyroptosis, metformin, NLRP3, SIRT1

## Abstract

Acute respiratory distress syndrome (ARDS), a devastating complication of numerous conditions, is often associated with high mortality. It is well known that endothelial cell (EC) damage and inflammation are vital processes in the pathogenesis of ARDS. Nevertheless, the mechanisms of EC damage are largely unknown. In the present study, we investigated the role of pyroptosis in the initiation of ARDS and demonstrated that endothelial pyroptosis might play a pivotal role in the pathophysiology of ARDS. Metformin, an antidiabetic drug, exhibited a protective effect in lipopolysaccharide (LPS)-induced lung injury, and we hypothesized that metformin alleviated LPS-induced lung injury *via* inhibiting ECs pyroptosis. *In vivo*, male ICR mice were intratracheally injected with LPS, and metformin was previously administered intraperitoneally. Morphological properties of lung tissues were detected. We showed that metformin inhibited NLRP3 inflammasome activation and NLRP3-stimulated pyroptosis induction, as shown by decreased levels of cleaved caspase-1, N-terminal fragment of GSDMD, and protein contents of IL-1β in lung tissues of mice exposed to LPS. LPS-induced expression of vascular adhesion molecules was also reduced after the treatment with metformin. *In vitro*, exposure of pulmonary ECs to LPS resulted in increased expression of NLRP3 and pyroptosis-associated indicators. By inhibiting the expression of NLRP3 with NLRP3 inhibitor MCC950, pyroptosis-related markers and vascular adhesion molecules were ameliorated. Moreover, metformin treatment significantly inhibited the NF-κB signaling pathway and increased the expression of sirtuin 1 (SIRT1) both in LPS-stimulated lung tissues and pulmonary ECs. Administration of the selective SIRT1 inhibitor nicotinamide significantly reversed the protective effect of metformin against endothelial pyroptosis and lung injury in LPS-treated ECs and LPS-induced acute lung injury (ALI). Thus, these findings demonstrated that metformin alleviated LPS-induced ALI by inhibiting NF-κB-NLRP3–mediated ECs pyroptosis, possibly by upregulating the expression of SIRT1.

## Introduction

Acute lung injury (ALI) or acute respiratory distress syndrome (ARDS) is a devastating complication triggered by various factors (Han and Mallampalli, 2015), and it is often associated with high mortality. It is characterized by difficult breathing and low blood oxygen, thereby leading directly to respiratory failure and death in fatal COVID-19 cases (Zhang et al., 2020). ARDS and sepsis share some of the underlying mechanisms, which include inflammatory responses and endothelial damage. Lung vascular endothelium injury is the most important initial cause of ARDS following sepsis, which affects pulmonary homeostasis (Matthay and Zemans, 2011). At present, advances in rescue therapies, including lung-protective ventilation (Amato et al., 2015), prone positioning (Guerin et al., 2013), infusion of neuromuscular blockade (Papazian et al., 2010), and extracorporeal life support (Peek et al., 2009), have improved the outcome of ARDS in recent years. However, there is no specific treatment strategy for ARDS. Therefore, further studies are needed to explore the pathogenesis and treatment modalities of ARDS.

Pyroptosis is a specialized form of cell death, different from apoptosis and necrosis (Shi et al., 2015). Canonical pyroptosis can be activated by inflammasomes (Jia et al., 2019). NLRP3 inflammasome can recognize microbial and endogenous danger signals and trigger innate immune responses. After priming, cytosolic NLRP3 oligomerizes with other proteins of the inflammasome complex, primarily ASC and pro-caspase-1 (Broz, 2019), and pro-caspase-1 is subsequently activated, leading to the cleavage of gasdermin D (GSDMD). After the activation, the N-terminal fragment of GSDMD oligomerizes and forms membrane pores, thereby causing cellular swelling, plasma membrane rupture, and release of cytoplasmic contents. Activation of caspase-1 also cleaves proinflammatory cytokines IL-1β and IL-18 into their functional conformation (Herman and Pasinetti, 2018; Mathur et al., 2018). Lung endothelial cells (ECs) are an important source of IL-1β, and the production of active IL-1β is controlled by the inflammasome. Pyroptosis has been shown to play a requisite role in endotoxemic lung injury, suggesting that inhibiting endothelial pyroptosis may offer an important therapeutic strategy for the treatment of ALI (Cheng et al., 2017).

Metformin is an effective antidiabetic drug which exhibits pulmonary protective effects in diverse ALI models, including ventilator-induced lung injury (Tsaknis et al., 2012), PM 2.5–induced lung injury (Gao et al., 2020), and endotoxemia-induced lung injury (Wu et al., 2018). In addition, metformin decreases the disease severity and mortality in COVID-19 patients (Bramante et al., 2021). Current evidence suggests that metformin alleviates endothelial dysfunction in the vasculature (de Aguiar et al., 2006). Metformin also protects from vascular endothelial dysfunction in type 2 diabetes patients with metabolic syndrome (Nafisa et al., 2018) and women with polycystic ovary syndrome (PCOS) (Heidari et al., 2019). NLRP3 inflammasome activation in lung vascular ECs is associated with different pulmonary disorders (Xiang et al., 2011; Wang et al., 2017; Ito et al., 2020). A previous study has demonstrated that metformin treatment ameliorates the NLRP3 inflammasome-mediated pyroptosis in diabetic periodontitis (Zhou et al., 2020); however, it remains unclear whether metformin reverses pulmonary endothelial dysfunction during the pathogenesis of ARDS.

Sirtuin 1 (SIRT1) is an NAD+ -dependent protein deacetylase that is involved in a wide range of cellular processes, including cellular metabolism and embryogenesis (Wang et al., 2008; Tang et al., 2014). There is emerging evidence that SIRT1 regulates NLRP3 in vascular ECs, thereby inhibiting the inflammatory response (Li et al., 2016; Li et al., 2017; Wang et al., 2017). NF-κB, a proinflammatory gene, activates a variety of genes in the nucleus, including NLRP3. Therefore, the present study investigated the effect of metformin in NLRP3 inflammasome activation-mediated pyroptosis exposed to lipopolysaccharide (LPS) and in LPS-treated lung ECs. Here, we demonstrated that metformin blunted the severity of LPS-induced ARDS and inflammasome activation. We hypothesize that metformin exerts the pulmonary protective role by upregulating the expression of SIRT1, thereby inhibiting NF-κB-NLRP3–triggered ECs pyroptosis. Therefore, we suggest that metformin, a safe and inexpensive antidiabetic drug, may be useful for the treatment of ARDS.

## Materials and Methods

### Animals and Experimental Design

Institute of Cancer Research (ICR) mice (25–30 g) were purchased from Shanghai Sippr-BK laboratory animal Co. Ltd. (Shanghai, China). The mice were acclimated to standard conditions (22 ± 2°C; 12 h light/dark periods) with access to food and water *ad libitum* for 7 days prior to conducting experiments. All animal studies were implemented in accordance with the principles of the Ethical Committee of Xinhua Hospital affiliated with Shanghai Jiao Tong University, School of Medicine. In addition, LPS from *Escherichia coli* O111:B4 (L2630, Sigma-Aldrich, St. Louis, MO, United States, 5 mg/kg) was intratracheally injected *via* a 22-gauge catheter, and the mice were euthanized 9 h later. Metformin (Bristol Myers Squibb, Shanghai, China) was administered intraperitoneally, 50 mg/kg, 30 min prior to the LPS challenge (Park et al., 2012; Vaez et al., 2016). SIRT-1 inhibitor nicotinamide (NAM, 60 mg/kg, Sigma) was given intraperitoneally 1 h before metformin treatment. At the indicated time, the mice were sacrificed, and the left lower lung lobes were removed and fixed in 4% formalin for morphological evaluation. Other lobes were frozen at −80°C for further analysis.

### Pulmonary ECs Cells Culture and Treatment

Mouse lung vascular ECs were isolated in accordance with a previously published article (Dong et al., 2015). In brief, ICR mice weighing around 15 g were anesthetized; their lungs were inflated with cold phosphate buffer saline (PBS) to remove blood; peripheral tissues were cut into small pieces. Dulbecco’s modified Eagle’s medium (DMEM) with 20% fetal bovine serum (FBS) was added as a culture medium. ECs crawled out beneath the lung tissue 60 h later. The adherent cells were grown in DMEM supplemented with 10% FBS for 3 days after removal of the tissue dices. Mouse lung ECs were characterized by their cobblestone morphology, and factor VIII-related antigen staining and CD31 staining were performed to identify their purity. The cells were exposed to 1 μg/ml LPS with/without pretreatment with metformin (10 mM), MCC950 (10 μM), NAM (1 mM), or SIRT1 siRNA (sc-40987, Santa Cruz). The supernatants were collected and stored at −80°C until assayed.

### Monocyte–ECs Adhesion

For activation, HMEC-1 cells (human microvascular endothelial cell-1) were cultured in 24-well plates and incubated with LPS and metformin in the presence or absence of NAM for 24 h. For monocyte–ECs adhesion experiments, assays were performed by adding 5  ×  10^5^ THP1 cells (Zhong Qiao Xin Zhou Biotechnology Co., Ltd., Shanghai, China) that were labeled with 2 µM calcein-AM with green fluorescence (Beyotime Biotechnology, China) to 1  ×  10^5^ confluent HMEC-1 cells (FuHeng Biology, Shanghai, China) for 2  h (Lin et al., 2018). THP-1 cells that were firmly bound to HMEC-1 were observed with a fluorescence microscope (Olympus, Tokyo, Japan).

### Histological Evaluation

Fixed lung specimens were embedded in paraffin, and tissue sections (4 μm) were stained with hematoxylin and eosin (H&E) to detect pathological changes.

### Lung Wet-To-Dry Ratio

The extent of lung edema was evaluated by the wet-to-dry (W/D) ratio. The wet weight was measured immediately after the excision of the lung, and the dry weight was recorded after drying in an oven at 60°C for 48 h. The lung W/D ratio was calculated as wet weight/dry weight.

### Evans Blue Albumin

An Evans blue dye–labeled albumin mixture (EB, 40 mg/kg, Sigma) was injected into the left jugular vein of each mouse 45 min before the mice were killed. The lungs were perfused with PBS and weighed. The lung tissues were homogenized in PBS and incubated with 2 ml formamide at 60°C overnight. The homogenate was centrifuged for 30 min at 5,000 g. The optical density of the supernatant was observed at 620 nm (A620) spectrophotometrically. Extravasated EB concentrations in the lung homogenate were presented as micrograms of Evans blue dye per gram of tissue.

### Immunohistochemistry and Immunofluorescence of Lung Samples

In brief, lung sections (4 μm) were deparaffinized, hydrated, and transferred to citrate buffer (pH = 6.0) for antigen retrieval, followed by cooling for 20 min. Endogenous peroxidase activity was blocked by H_2_O_2_, and incubation with normal goat serum was performed to reduce nonspecific binding. The lung sections were incubated with primary antibodies specific for F4/80 (a macrophage marker) and myeloperoxidase (MPO, a neutrophil marker). NLRP3 and caspase-1 activation in pulmonary endothelium was detected by immunofluorescence co-staining with the endothelial marker CD31 in accordance with standard protocols. The sections were imaged through an Olympus fluorescence microscope.

### Immunofluorescence Staining of Endothelial Cells

After the treatments, endothelial cells were fixed with 4% paraformaldehyde for 10–20°min, permeabilized with 0.3% Triton X-100 in PBS-Tween, and blocked with 5% BSA for 30°min, followed by incubation with primary antibody against NLRP3 (1:200, ABclonal) or CD31 (1:200, Servicebio) at 4°C overnight. FITC-conjugated or CY3-conjugated secondary antibodies were used for immunodetection. After washing three times, nuclei were stained with 4′,6-diamidino-2-phenylindole (DAPI, Beyotime) for 5 min. Then, the cells were observed, and images were captured under a fluorescence microscope (Olympus).

### TUNEL Assay

DNA fragmentation of endothelial cells was measured by TdT-mediated dUTP nick-end labeling (TUNEL) staining in line with the manufacturer’s protocol (Beyotime). In brief, endothelial cells were fixed and permeabilized, as described earlier. After rinsing with PBS, the cells were incubated with a one-step TUNEL reaction mixture for 1 h at 37°C in a humidified chamber in the dark. Finally, nuclei were counterstained with DAPI (Beyotime). Images were observed, and photographs were taken under a fluorescence microscope (Olympus).

### Western Blot Analysis

Lung samples and cell lysates were prepared in cold RIPA buffer containing a protease inhibitor cocktail (Sigma). Equal amounts of proteins (40 μg) were separated by SDS-PAGE and then transferred to PVDF membranes. The membranes were blocked with 5% nonfat milk and incubated with the following primary antibodies: anti-NLRP3 (1:1,000, Cell Signaling Technology, CST, Inc., United States), anti-caspase-1 (1:1,000, ABclonal), anti-GSDMD (1:500, Abcam, Cambridge, MA, United States), anti-SIRT1 (1:1,000, CST), anti-NF-κB p65 (1:1,000, CST), anti-phosphorylated (p)-NF-κB p65 (1:1,000, CST), anti-IκB-α (1:1,000, CST), anti-Acetyl-NF-κB p65 (Lys310) (1:500, CST), anti-PCNA (D3H8P) (1:500, CST), anti-ICAM-1 (1:1,000, Servicebio), and anti-VCAM-1 (1:1,000, Servicebio) overnight at 4°C. After that, the membranes were incubated with HRP-conjugated secondary antibody for 1–2 h. The blots were detected with an enhanced chemiluminescence (ECL) system (Thermo Fisher Scientific). The protein band intensity was normalized to β-actin (1:3,000, Sigma) and expressed as a ratio of the control.

### Nuclear and Cytoplasmic Protein Extraction

The nuclear and cytoplasmic protein extraction was conducted by following the manufacturer’s instructions of the commercial kit (P0028, Beyotime).

### ELISA

The concentrations of IL-1β in lung tissues and cell supernatants were determined by enzyme-linked immunosorbent assay (ELISA) kits in accordance with the manufacturer’s instructions (Elabscience Biotechnology Co., Ltd., Wuhan, China).

### Statistical Analysis

All data are expressed as the mean ± standard error of mean (SEM). Differences among different groups were analyzed by one-way analysis of variance (ANOVA), followed by a *post-hoc* Tukey’s multiple-comparison test using SPSS software. The significance level was set at *p* < 0.05.

## Results

### Metformin Ameliorates LPS-Induced Lung Injury in Mice

**FIGURE 1:**
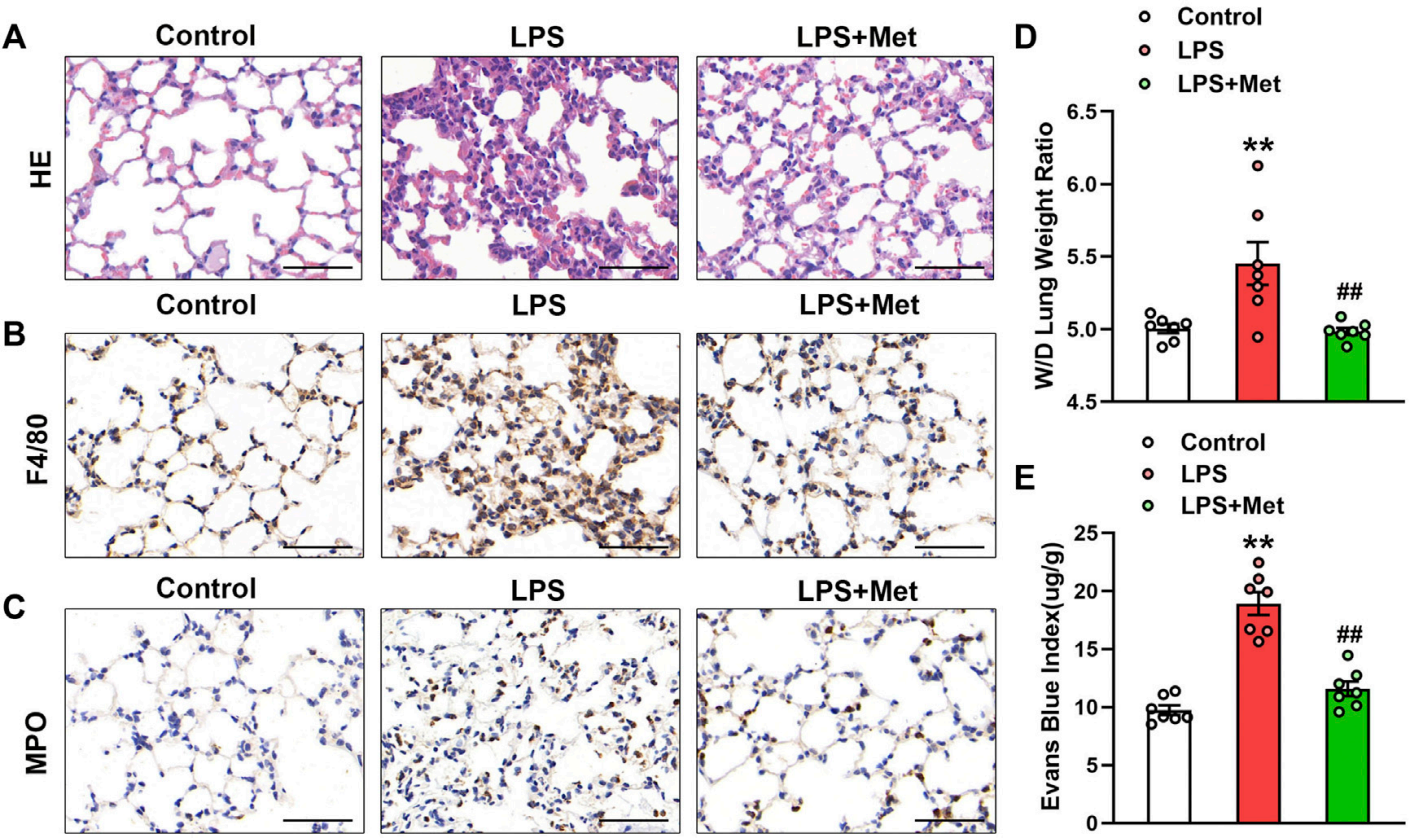
Metformin ameliorates the morphological characteristics and vascular endothelial hyperpermeability of LPS-induced ALI in mice. Mice were subjected to LPS, with or without metformin treatment for 9 h. **(A)** Representative hematoxylin and eosin staining of the lung tissues. **(B)** Immunohistochemistry analysis was used to measure the intensity of F4/80. **(C)** Immunohistochemistry was used to measure the intensity of myeloperoxidase. **(D and E)** Lung W/D and Evans blue dye extravasation were determined as indexes of pulmonary edema. Original magnification, ×200. Scale bar, 50 μm. Data are presented as the mean ± SEM (*n* = 7). **p < 0.01 vs. Control group, ##*p* < 0.01 vs. LPS group. Myeloperoxidase, MPO.

To examine the effects of metformin on LPS-induced ALI, we assessed morphological characteristics, performed immunohistochemical analysis of F4/80 and MPO in lung tissues of mice to show the changes in terms of inflammation and neutrophils, and evaluated lung W/D and EBA to determine pulmonary endothelial permeability. Compared with the control group, LPS administration resulted in severe lung injury; in contrast, metformin pretreatment significantly improved LPS-induced lung injury, as reflected by decreased intra-alveolar and interstitial edema (Figure 1A). Metformin also strongly inhibited infiltration by macrophages and neutrophils (Figures 1B and C). LPS administration damaged the pulmonary vascular endothelium as evidenced by increased W/D ratio and degree of EBA extravasation, but these effects were also reduced by metformin (Figures 1D and E).

### Metformin Suppresses LPS-Induced Endothelial Cell Pyroptosis and Vascular Adhesion Molecules *In Vivo*

**FIGURE 2:**
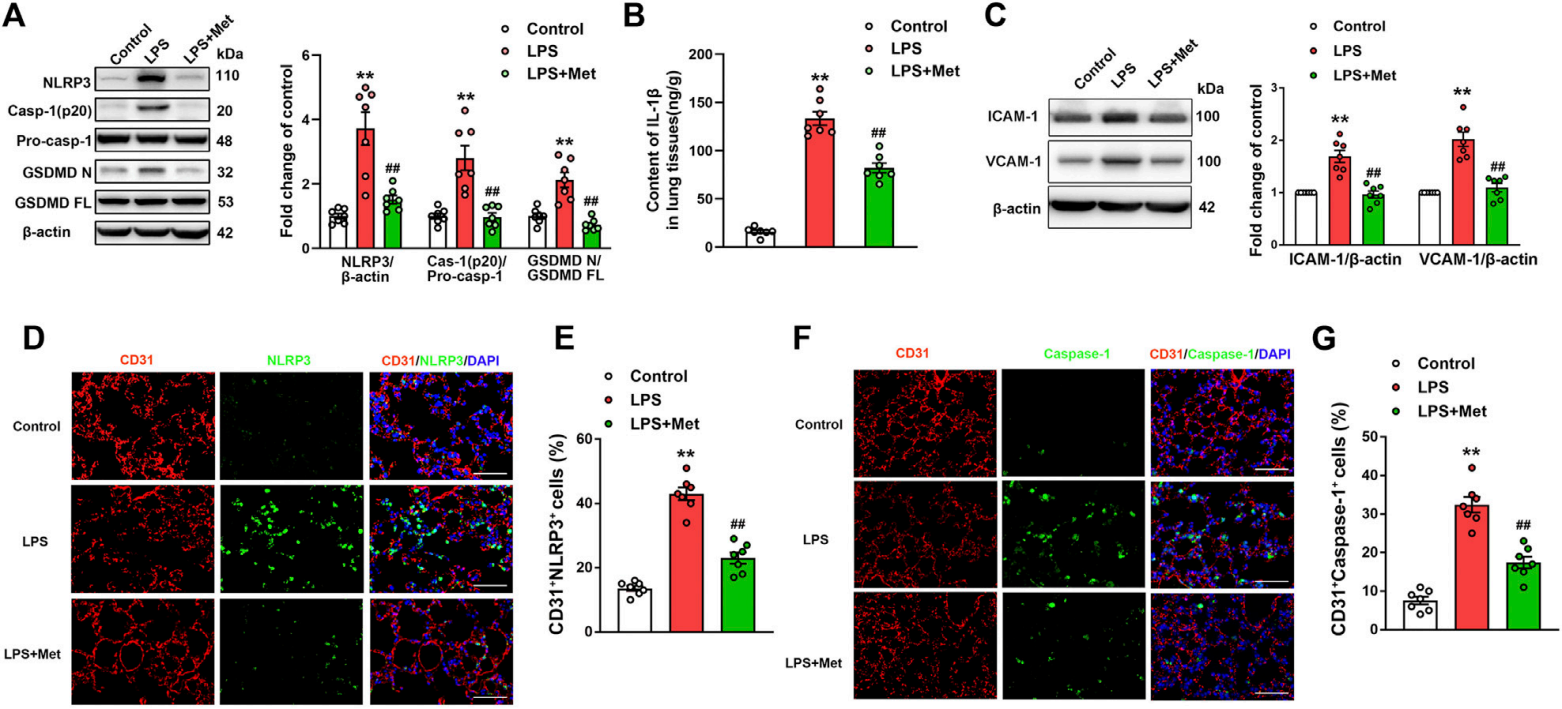
Metformin reduces the LPS-induced release of pyroptotic proteins and vascular adhesion molecules in mouse lung tissues. **(A)** Western blotting was used to determine the levels of NLRP3, cleaved caspase 1 (p20), and N-terminal fragment of GSDMD. Indicative bands are shown. **(B)** ELISA was used to determine the protein levels of IL-1β. **(C)** Representative protein bands and corresponding histogram of ICAM-1 and VCAM-1 are shown. **(D)** Lung tissues were stained with NLRP3 (green) and CD31 (red). Nuclei were counterstained with 4’6-diamidino-2-phenylindole (DAPI) (blue). **(E)** Percentages of CD31^+^NLRP3^+^ cells are shown as histograms. **(F)** Lung tissues were stained with caspase-1 (green) and CD31 (red). Nuclei were counterstained with DAPI (blue). **(G)** Percentages of CD31^+^Caspase-1^+^ cells are shown as histograms. Original magnification, ×200. Scale bar, 50 μm. Data are presented as the mean ± SEM (*n* = 7). **p < 0.01 vs. Control group, ##*p* < 0.01 vs. LPS group.

To investigate whether the mitigation of lung injury by metformin was associated with pyroptosis, the expression levels of pyroptosis-related proteins were detected. As expected, the protein levels of NLRP3, activated caspase-1 (caspase-1 p20), and the N-terminal fragment of GSDMD and IL-1β level were significantly increased in lung tissues of mice exposed to LPS, and metformin pretreatment significantly decreased the levels of these pyroptosis-related proteins (Figures 2A and B). The results showed that the administration of LPS caused a significant increase in the expression of vascular adhesive molecules, including ICAM-1 and VCAM-1, while metformin pretreatment significantly decreased the expression levels of ICAM-1 and VCAM-1 (Figure 2C). To further demonstrate that pyroptosis occurred in pulmonary endothelium, CD31/NLRP3 and CD31/caspase-1 double staining were performed. As shown in Figures 2D–G, the percentage of NLRP3- and caspase-1-positive cells was markedly increased in the pulmonary endothelium of lung tissues following the LPS challenge, but the supplementation of metformin significantly decreased active NLRP3 and caspase-1-positive cells. Collectively, these results showed that metformin inhibited pulmonary EC pyroptosis and vascular adhesive molecules during LPS-stimulated lung injury.

### Metformin Suppresses LPS-Induced Endothelial Cell Pyroptosis *In Vitro*

**FIGURE 3:**
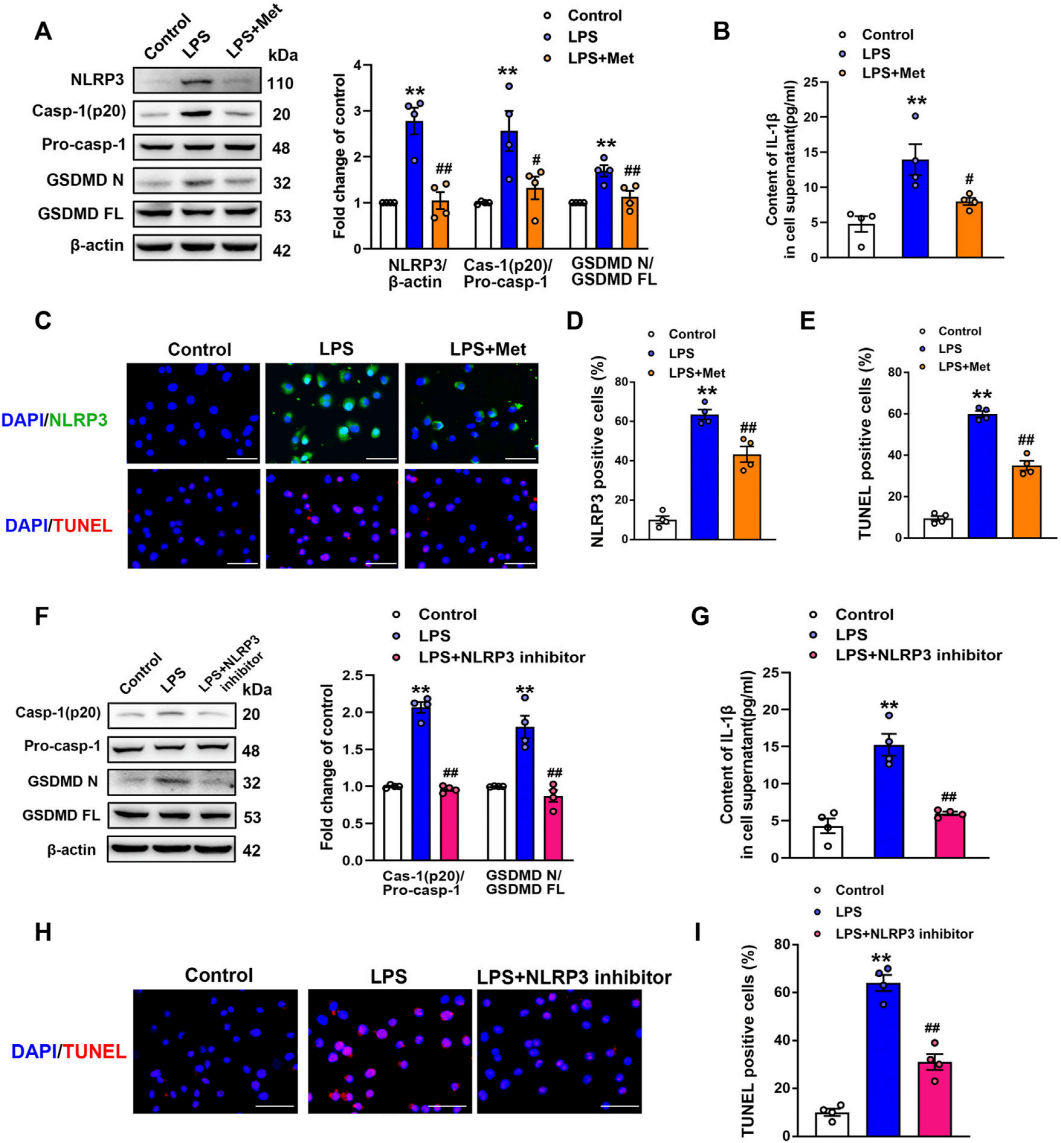
Metformin and NLRP3 inhibitor reduce the LPS-induced release of pyroptotic proteins and inflammatory cytokines in primarily cultured pulmonary ECs of mice. Pulmonary ECs were pretreated with metformin (10 mM) and NLRP3 inhibitor (MCC950, 10 μM), and then ECs were exposed to LPS for 24 h. **(A)** Indicative protein bands of NLRP3, cleaved caspase 1 (p20), and N-terminal fragment of GSDMD. **(B)** ELISA was used to determine the protein levels of IL-1β in the supernatant. **(C)** ECs were stained with NLRP3 (green). Apoptotic cells were evaluated using TUNEL staining. The nuclei were stained blue using DAPI. **(D)** and **(E)** Percentages of NLRP3-positive cells and TUNEL-positive cells. **(F)** Effect of MCC950 pretreatment on the expression levels of pyroptosis-related proteins was analyzed by Western blotting. Quantitative analysis of pyroptosis-related protein expression was conducted. Representative bands are shown on the left of the histogram. **(G)** ELISA was used to determine the protein levels of IL-1β in supernatants. **(H)** DNA fragmentation was determined using TUNEL staining. The nuclei were stained blue using DAPI. **(I)** Percentage of TUNEL-positive cells. Magnification: ×200. Scale bar, 50 μm. Data are presented as the mean ± SEM (*n* = 4). **p < 0.01 vs. Control group, #*p* < 0.05, ##*p* < 0.01 vs. LPS group.

To further explore the role of metformin in LPS-induced endothelial injury, mouse lung ECs were exposed to LPS with or without metformin added. As shown in Figure 3A, the protein levels of NLRP3, cleaved caspase-1, and N-terminal fragment of GSDMD were increased in LPS-treated ECs. IL-1β in cell supernatant was also elevated after LPS administration (Figure 3B). Metformin pretreatment also inhibited the expression of ICAM-1 and VCAM-1 (Supplementary Figure S2). Furthermore, the immunofluorescence staining of NLRP3 and TUNEL staining, respectively, revealed that NLRP3-positive cells and apoptotic cells were remarkably increased by LPS treatment, whereas all these effects were attenuated after pretreatment with metformin (Figures 3C–E).

### NLRP3 Inflammasome Induces Pyroptosis Activation in LPS-Treated ECs

NLRP3 inflammasome is well known for its involvement in the activation of caspase-1 and subsequent pyroptosis (Wang et al., 2019). To confirm our hypothesis, NLRP3 inhibitory experiments with the NLRP3 inhibitor MCC950 were performed. Notably, the results showed that MCC950 treatment significantly inhibited the LPS-induced increases in the protein levels of activated caspase-1 (p20) and N-terminal fragment of GSDMD, and the LPS-induced release of IL-1β was also suppressed by MCC950 treatment (Figures 3F and G). The expression levels of vascular adhesion molecules ICAM-1 and VCAM-1 were equally diminished following MCC950 treatment (Supplementary Figure S3). Moreover, the percentages of TUNEL-positive cells were reduced after the treatment with MCC950 in LPS-treated ECs (Figures 3H and I). Therefore, these data provided strong evidence that ECs pyroptosis in LPS-stimulated lung injury was mediated by NLRP3 inflammasome activation.

### Effects of Metformin on the Expression of SIRT1/NF-κB Pathway *In Vivo* and *In Vitro*

**FIGURE 4:**
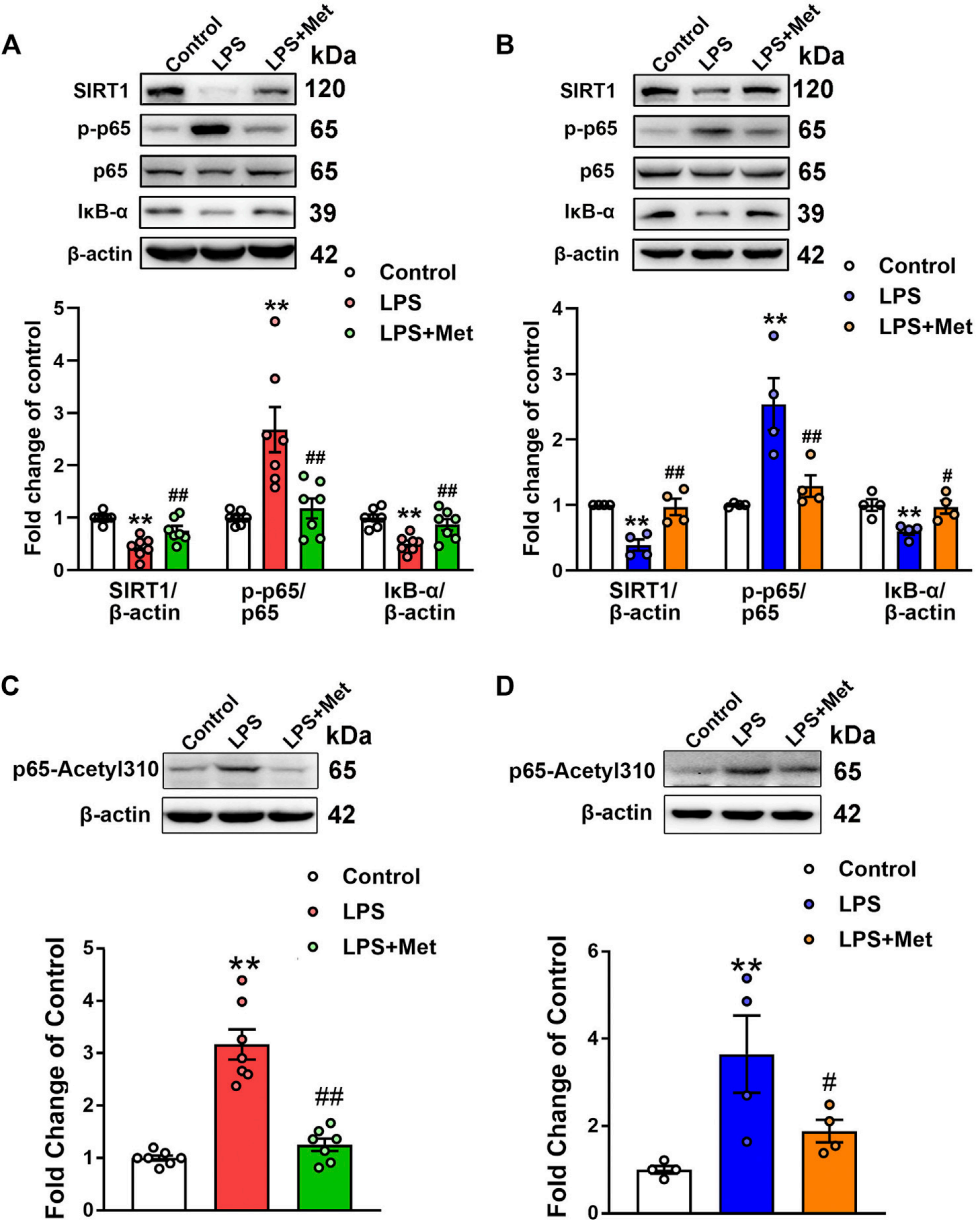
Metformin suppresses the LPS-induced downregulation of SIRT1 both *in vivo* and *in vitro*. **(A)** Effects of metformin on the expression level of SIRT1 and NF-κB signaling pathway in pulmonary tissues of mice that received LPS intratracheally. Indicative bands are shown on the top of the histogram. Data are presented as mean ± SEM (*n* = 7). **(B)** Effects of metformin on the expression level of SIRT1 and NF-κB signaling pathway in primarily cultured pulmonary ECs. Representative bands are shown. Data are presented as mean ± SEM (*n* = 4). **(C)** Effects of metformin on the expression level of p65 lysine 310 acetylation in LPS-challenged mouse lung tissues. Data are presented as mean ± SEM (*n* = 7). **(D)** Effects of metformin on the expression level of p65 lysine 310 acetylation in LPS-treated pulmonary ECs. Data are presented as mean ± SEM (*n* = 4). **p < 0.01 vs. Control group, #*p* <0.05, ##*p* < 0.01 vs. LPS group.

Subsequently, we assessed the effects of metformin on the protein levels of SIRT1 in the lungs of the mouse ARDS model and LPS-treated pulmonary ECs. Western blot analysis indicated that the relative level of SIRT1 in the LPS group decreased to 43% of the control level, whereas further treatment with metformin increased it to nearly 75% of the control level (Figure 4A). Similarly, in pulmonary ECs exposed to LPS, the protein level of SIRT1 was decreased, while metformin administration restored the expression level of SIRT1 (Figure 4B). Phosphorylated NF-κB p65 subunit was increased, whereas IκB-α was decreased in the LPS group compared with the control group; however, metformin reversed these effects both *in vivo* (Figure 4A) and *in vitro* (Figure 4B). These results indicated that metformin increased the expression of SIRT1 and decreased the level of phosphorylated NF-κB p65. It has been reported that SIRT1 reduces NF-κB activity by decreasing the acetylation level of lysine 310 of the NF-κB p65 subunit; as expected, the enhancement of p65 lysine 310 acetylation after LPS administration was detected, but it decreased after metformin treatment both *in vivo* and *in vitro* (Figures 4C and D).

### Inhibition of SIRT1 Eliminates the Protective Effect of Metformin on Endothelial Cell Pyroptosis *In Vitro*

**FIGURE 5:**
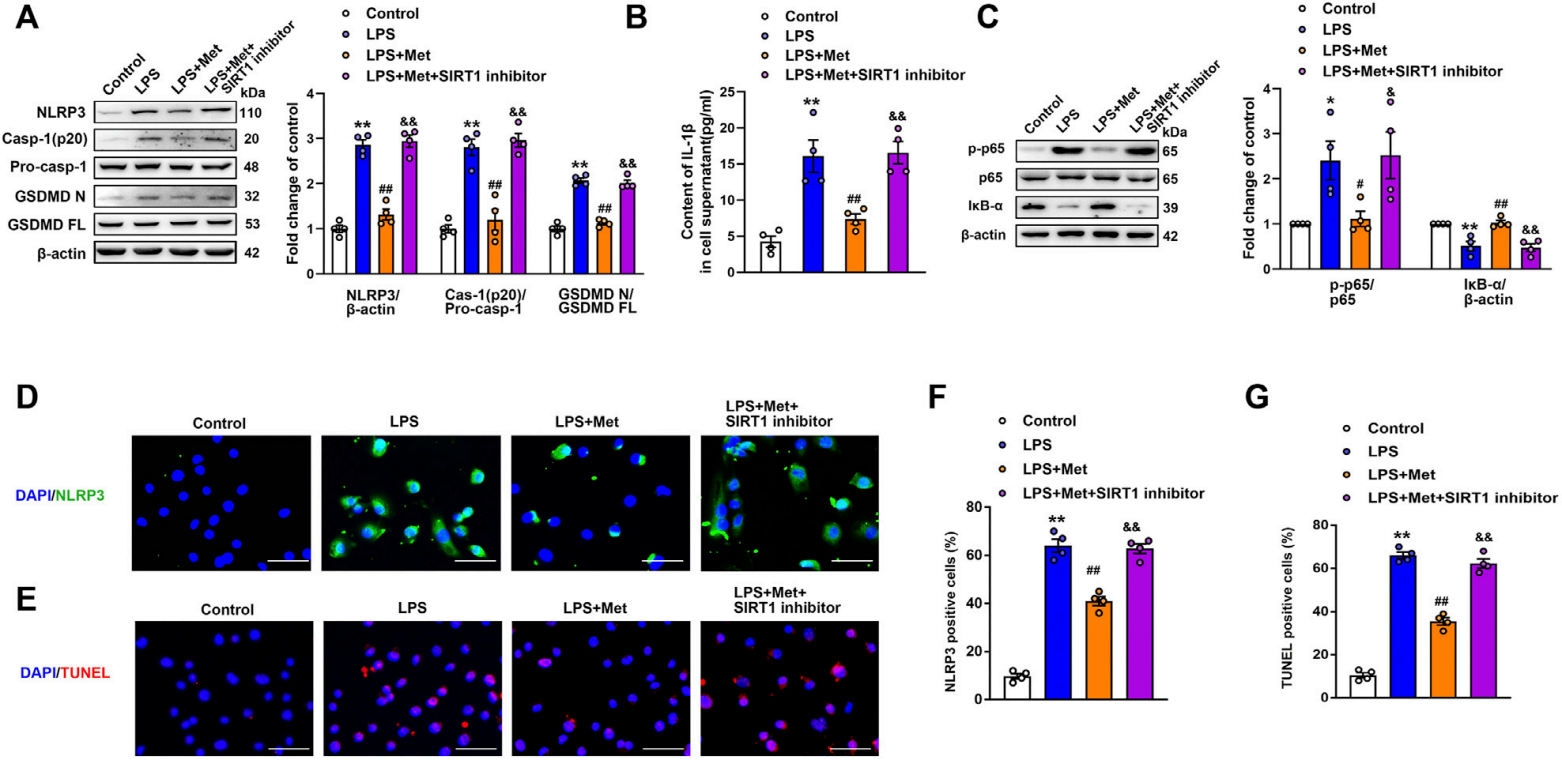
The SIRT1 inhibitor nicotinamide prevents metformin from reducing LPS-induced pyroptosis in mice pulmonary ECs. **(A)** Western blotting analysis was used to determine the levels of NLRP3, cleaved caspase 1 (p20), and N-terminal fragment of GSDMD. Indicative bands are shown on the left of the histogram. **(B)** ELISA was used to determine the protein levels of IL-1β in supernatants. **(C)** Representative protein bands of NF-κB signaling pathway after pretreatment with NAM. **(D)** ECs were stained with NLRP3 (green). **(E)** Apoptotic cells were evaluated using TUNEL staining. The nuclei were stained blue using DAPI. **(F and G)** Percentage of NLRP3- and TUNEL-positive cells. Magnification: ×200. Scale bar, 50 μm. Data are presented as the mean ± SEM (*n* = 4). *p < 0.05, **p < 0.01 vs. Control group, #*p* < 0.05, ##*p* < 0.01 vs. LPS group. &*p* < 0.05, &&*p* < 0.01 vs. LPS + Met group.

A former study demonstrated that the activation of SIRT1 inhibited NLRP3 inflammasome activation and subsequent caspase-1 cleavage and IL-1β secretion (Li et al., 2017). In our experiment, we examined whether the inhibition of NLRP3 inflammasome mediated by metformin was regulated by SIRT1. The ECs were incubated with the SIRT1 inhibitor NAM before LPS and metformin treatment. Western blotting indicated that the protein levels of NLRP3, cleaved caspase-1, and N-terminal fragment of GSDMD were increased in the LPS group; however, these proteins decreased in the LPS + Met group and increased again in the LPS + Met + NAM group (Figure 5A). Furthermore, ELISA indicated that the suppression of SIRT1 reversed the effects of metformin on IL-1β levels in LPS-treated pulmonary ECs (Figure 5B). Similarly, treatment with NAM blocked the protective effect of metformin on LPS-induced expression of vascular adhesion molecules (Supplementary Figure S4). Phosphorylated NF-κB p65 subunit was increased, and IκB-α was decreased in the LPS + Met + NAM group compared with the LPS + Met group (Figure 5C). Moreover, p65 lysine 310 acetylation was increased after SIRT1 inhibitor treatment (Supplementary Figure S5B). Meanwhile, SIRT1 siRNA was used to specifically knockdown the expression of SIRT1 in mouse pulmonary ECs; NF-κB p65 was assessed in nuclear lysates and cytoplasm, and representative bands are shown in Supplementary Figure S6. Furthermore, NLRP3 fluorescence intensity and TUNEL-positive endothelial cells, which were attenuated after treatment with metformin, were reversed by NAM administration (Figures 5D–G).

### Inhibition of SIRT1 Reverses the Anti-Pyroptosis and Anti–Vascular Adhesion Molecules Effects of Metformin in Mice With LPS-Induced ALI

**FIGURE 6:**
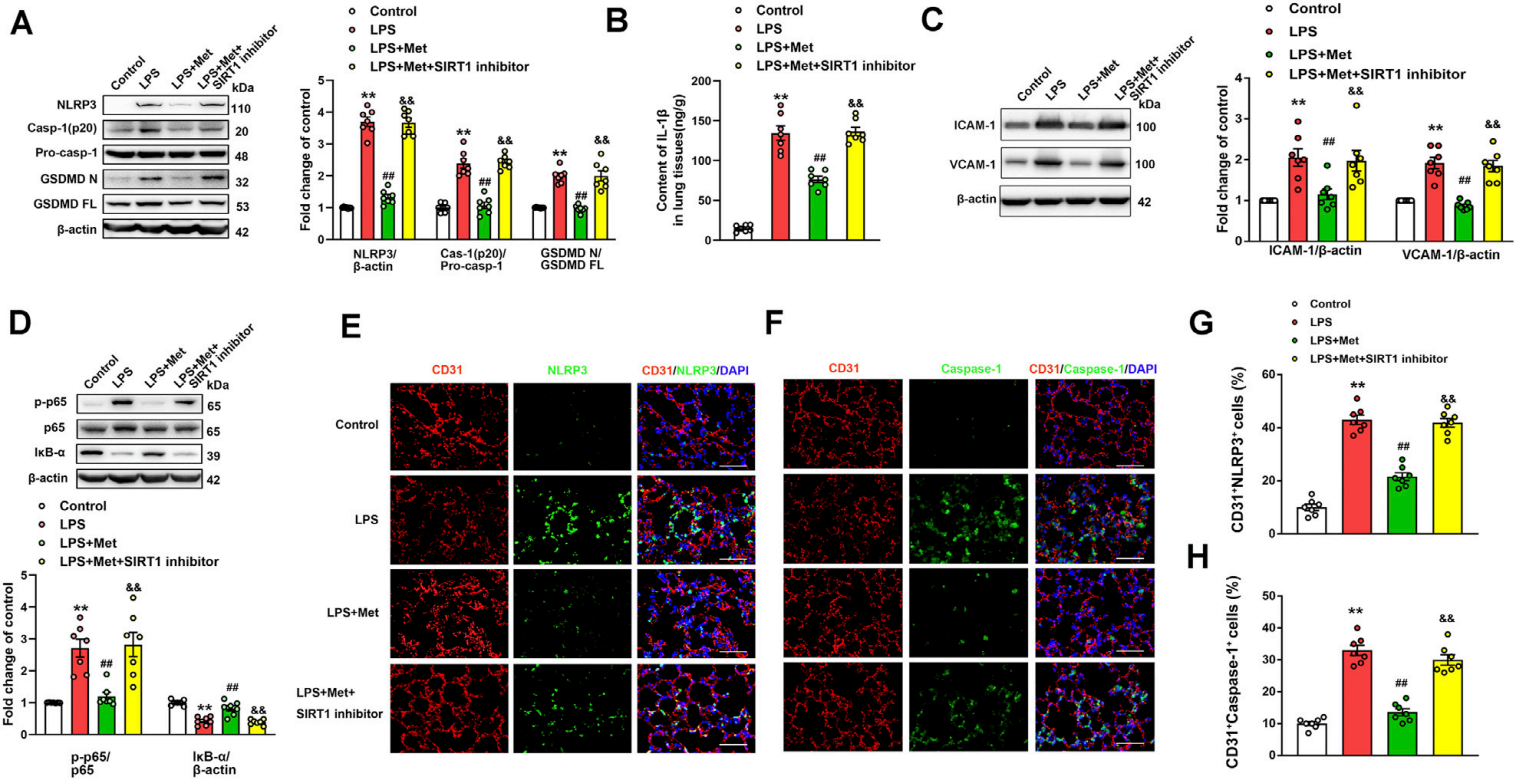
The inhibition of SIRT1 prevents metformin from suppressing pyroptosis and vascular adhesion molecules in the lungs of LPS-induced ALI mice. **(A)** Represented pyroptosis-associated proteins in lung tissues of LPS-induced ALI mice after administration of NAM. **(B)** IL-1β protein level was detected. **(C)** Representative protein bands of ICAM-1 and VCAM-1 in lung tissue. **(D)** Representative protein bands of the NF-κB signaling pathway in lung tissues after pretreatment with NAM. **(E)** Lung tissues were stained with NLRP3 (green) and CD31 (red). Nuclei were counterstained with 4’6-diamidino-2-phenylindole (DAPI) (blue). **(F)** Lung tissues were stained with caspase-1 (green) and CD31 (red). Nuclei were counterstained with DAPI (blue). **(G)** Percentage of CD31^+^NLRP3^+^ cells. **(H)** Percentage of CD31^+^Caspase-1^+^ cells. Original magnification, ×200. Scale bar, 50 μm. Data are presented as the mean ± SEM (*n* = 7). **p < 0.01 vs. Control group, ##*p* < 0.01 vs. LPS group. &&*p* < 0.01 vs. LPS + Met group.

The role of the SIRT1 inhibitor in the anti-pyroptosis and anti–vascular adhesion molecules effects of metformin in LPS-induced ALI mice was further verified. As shown in Figure 6A, metformin significantly inhibited the expression levels of pyroptosis-associated proteins, including NLRP3, caspase-1 p20 fragment, and GSDMD p30 fragment, in the lung tissues of the mice challenged with LPS. However, when NAM was used to inhibit SIRT1, the anti-pyroptosis effects of metformin were suppressed. NAM also reversed the effects of metformin on IL-1β levels in the lungs of the mice exposed to LPS (Figure 6B). We also detected the protein levels of vascular adhesion molecules ICAM-1 and VCAM-1; the results indicated that after treatment with NAM, metformin no longer showed an inhibitory effect on vascular adhesion molecules (Figure 6C). The lung tissue level of the phosphorylated NF-κB p65 subunit was increased, whereas that of IκB-α was decreased in the LPS + Met + NAM group compared with the LPS + Met group (Figure 6D). The enhancement of p65 lysine 310 acetylation post-NAM treatment was also detected (Fig. S5A). CD31^+^ NLRP3^+^ and CD31^+^ caspase-1^+^ cells were markedly reduced in lung tissues of ALI mice after treatment with metformin, and treatment with NAM reversed the inhibitory effect of metformin on NLRP3 and caspase-1 activation in the pulmonary endothelial cells of mice exposed to LPS administration (Figures 6E–H).

### Inhibition of SIRT1 Eliminates the Protective Effect of Metformin on LPS-Induced Lung Injury in Mice

**FIGURE 7:**
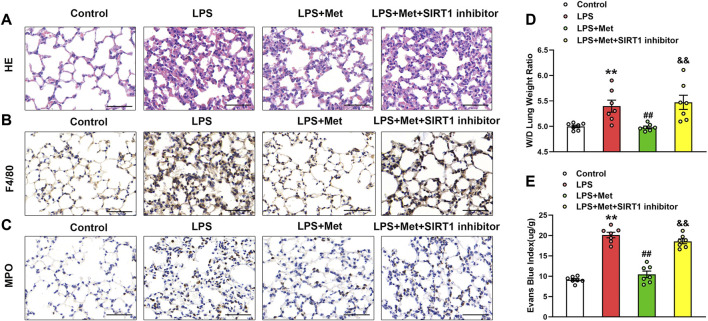
The inhibition of SIRT1 prevents metformin from ameliorating the morphological characteristics and endothelial hyperpermeability of ALI. NAM (60 mg/kg) was administered 1 h before metformin administration. **(A)** Representative HE staining of lung tissues. **(B)** Immunohistochemistry analysis of F4/80. **(C)** Immunohistochemistry of MPO. **(D and E)** Lung W/D and Evans-blue dye extravasation were determined as indexes of pulmonary edema. Original magnification, ×200. Scale bar, 50 μm. Data are presented as the mean ± SEM (*n* = 7). **p < 0.01 vs. Control group, ##*p* < 0.01 vs. LPS group. &&*p* < 0.01 vs. LPS + Met group.

The SIRT1 inhibitor was used to evaluate the protective role of metformin on LPS-induced lung injury. As shown in Figures 7A–C, the treatment with NAM reversed the protective effects of metformin on LPS-stimulated lung injury, macrophage, and neutrophil infiltration. The W/D ratio and EBA extravasation were also elevated in the LPS + Met + NAM group compared with the LPS + Met group (Figures 7D and E), indicating that SIRT1 was a crucial facilitator in the protective effect of metformin in the lungs of LPS-induced ALI mice.

## Discussion

In the current study, we illustrated that ECs pyroptosis plays a pivotal role in the pathogenesis of LPS-induced ARDS. We showed that the activation of pyroptosis was mediated by NF-κB, leading to elevated expression of NLRP3 and subsequent caspase-1 activation to produce and release inflammatory cytokines. Metformin, an antidiabetic drug, exhibited protective effects against LPS-induced ARDS *via* the activation of SIRT1, thereby inhibiting NF-κB/NLRP3 pathway–mediated pulmonary vascular ECs pyroptosis.

ARDS is a life-threatening lung injury of seriously ill patients, which is manifested by acute hypoxic respiratory failure and pulmonary inflammatory infiltrates (Diamond et al., 2021). A retrospective cohort study showed that metformin reduced mortality in COVID-19 patients in women with obesity or type 2 diabetes (Bramante et al., 2021). The protective effect of metformin may be attributed to its additional impact on inflammation (Valencia et al., 2017), given that the levels of high-sensitivity C-reactive protein after metformin treatment are reduced in women with diabetes compared with the placebo group (Haffner et al., 2005). Furthermore, short-term metformin treatment of non-diabetic subjects prevents acute inflammatory responses, such as ARDS. Researchers have demonstrated that metformin treatment prevents and ameliorates ARDS induced by LPS in mice, accompanied by abrogated NLRP3 inflammasome activation in macrophages, and attenuates pulmonary inflammation in COVID-19 patients (Xian et al., 2021). In the present study, we showed that metformin alleviated LPS-induced lung inflammation, including decreased MPO and macrophage infiltration. Notably, endothelial hyperpermeability in the lungs of LPS-induced ALI mice was also improved by metformin.

Metformin has been reported to improve vascular endothelial reactivity in type 2 diabetes patients with metabolic syndrome, which is a novel function in addition to its well-known antihyperglycemic effects (de Aguiar et al., 2006). Moreover, metformin protects against endothelial dysfunction in patients with metabolic syndrome by improving insulin resistance (Vitale et al., 2005). The effects of metformin in non-metabolic disease have also been studied. Current evidence suggests that concomitant administration of metformin during radiotherapy in rats decreases the expression of E-selectin, ICAM-1, and VCAM-1, thereby acting as a potent heart protector from endothelial dysfunction-induced damage (Karam and Radwan, 2019). There are multiple mechanisms by which metformin improves endothelial dysfunction, including the inhibition of an important mediator, LOX-1 signaling, thereby decreasing intracellular oxidative stress (Hattori et al., 2006; Xu et al., 2013). Other mechanisms underlying the protective effect of metformin in endothelial dysfunction also include inhibition of endothelial senescence and apoptosis (Arunachalam et al., 2014). In the present study, we found that metformin pretreatment effectively prevented pyroptosis in LPS-induced ARDS mice and LPS-treated lung ECs.

In eukaryotic cells, pyroptosis induced by inflammatory caspases serves as an innate immune strategy to protect against microbial infections (Bergsbaken et al., 2009; Jorgensen and Miao, 2015). Unlike the other forms of programmed cell death, here, the activation of caspases triggers lytic cell death, and GSDMD leads to pore formation, finally resulting in the cleavage of inflammatory cytokine IL-1β (Wallach et al., 2016). Unfettered pyroptosis can induce injury of multiple tissues, including ALI (Hou et al., 2018; Zeng et al., 2019); thus, targeting caspase-mediated pyroptosis in specific cell types, such as macrophages (Li et al., 2018), endothelial cells (Cheng et al., 2017), and epithelial cells (Zhang et al., 2018), may be a potential therapeutic strategy to alleviate inflammatory lung damage and organ failure, without degradation of the host defense barrier. Although the protective effects of metformin against lung injury have been well-demonstrated in various models (Tsaknis et al., 2012; Chen et al., 2015; Wu et al., 2018; Cheng et al., 2021), its protective effect against pulmonary ECs injury has not yet been reported. ECs line the surface of lung vasculature; thus, they are the first point of contact for pathogens and bacterial toxins and also the first point to respond to them. Vascular endothelial barrier refers to a semipermeable barrier that regulates the exchange of blood fluid and electrolytes across the blood vessel wall. At the blood–tissue interface, host defense is activated by an endothelial-based LPS detection system, which provides a defense mechanism. Therefore, the critical role of ECs in the systemic immune response to severe bacterial infection should be highlighted. Inflammasome and subsequent proinflammatory mediators release can be activated by administration of LPS into the lung tissue. It has been shown that LPS induces activation of inflammasome in mouse pulmonary ECs, thereby resulting in caspase-1 activation (Yang et al., 2016). In our study, mice lung tissues and ECs exposed to LPS showed increased expression levels of NLRP3 and cleaved GSDMD fragment and release of the proinflammatory cytokine IL-1β. Administration of metformin and NLRP3 inhibitor MCC950 remarkably decreased inflammasome activation and the level of pyroptosis-related indicators in LPS-treated pulmonary ECs and LPS-induced ALI mice, indicating that the potential therapeutic role of metformin in targeting the endothelial pyroptosis in ALI may be attributed to the activation of NLRP3 inflammasome. This is consistent with a recently published article that showed metformin abrogated macrophage NLRP3 inflammasome activation and pulmonary inflammation (Xian et al., 2021). Considering that metformin significantly inhibited macrophage inhibition, we detected the expression of vascular endothelial cell adhesion molecules ICAM-1 and VCAM-1 both *in vivo* and *in vitro*. We also examined the attachment of monocytes onto the activated endothelial cells. All these results suggested the underlying role of metformin in inhibiting macrophage infiltration.

K^+^ efflux, reactive oxygen species (ROS) release, and lysosomal disruption can activate the NLRP3 inflammasome (Sutterwala et al., 2014). In addition, NF-κB is a central mediator of the priming signal of NLRP3 inflammasome activation; namely, activated NF-κB is translocated from cytoplasm to nucleus, whereby it transcriptionally activates the secretion of pro-IL-1β, pro-IL-18, and NLRP3, which are crucial for the induction of pyroptosis in several diseases (Li et al., 2019). Hattori et al. (2006) showed that metformin inhibited the cytokine-induced expression of proinflammatory and adhesion molecule genes by blocking NF-κB activation in vascular endothelial cells *via* AMPK activation. In this study, metformin inhibited NF-κB phosphorylation in pulmonary tissues and mouse lung ECs following LPS treatment, indicating that metformin inhibited the NF-κB signaling pathway in LPS-induced pulmonary endothelial injury. SIRT1 has been reported to inhibit NF-κB by directly deacetylating the p65/RelA at lysine 310 (Yeung et al., 2004) or activating AMPK and PPARα, thus inhibiting the NF-κB pathway (Zhang et al., 2017). Notably, our study also demonstrated that the beneficial effects of metformin were mediated by SIRT1, as shown by the effects of the SIRT1 inhibitor NAM. Significant elevation of NF-κB phosphorylation and NLRP3-mediated pyroptosis-associated proteins due to inhibited SIRT1 expression by NAM was shown. Usually, NF-κB is bound in the cytoplasm by its inhibitor IκB. Upon stimulation, IκB is degraded so that NF-κB can translocate to the nucleus and stimulate inflammatory gene expression. In this study, SIRT1 inhibitors increased NF-κB p65 phosphorylation, showing that NF-κB p65 acts as a downstream target of SIRT1. Acetylation of the p65 at lysine 310 is a possible mechanism underlying the proinflammatory effect of the SIRT1 inhibitor (Schug et al., 2010). In this study, we also showed that SIRT1 reduced NF-κB activity by decreasing the acetylation level of lysine 310 of the NF-κB p65 subunit. However, we believe that it may not be the sole explanation because SIRT1 deletion inhibited the expression of IκB-α, which is an upstream signal of p65 nuclear translocation. Thus, further studies are warranted with regard to how SIRT1 regulates the NF-κB pathway.

In the present study, we observed elevated expression levels of NLRP3 and pyroptosis-associated proteins in LPS-treated endothelial cells and in the mouse lung tissues exposed to LPS. Furthermore, pyroptosis may play a crucial role in the development of LPS-induced inflammatory lung injury, whilst pulmonary endothelium is impaired and unable to provide barrier support effectively. Metformin recovered those detrimental changes, protected against endothelial dysfunction, and ultimately repaired the impaired lung functions, and the underlying mechanisms may be related to the activation of SIRT1. In summary, these results suggested that inhibition of endothelial pyroptosis and metformin treatment could be used as a novel therapeutic intervention strategy for ARDS treatment.

## Data Availability

The raw data supporting the conclusions of this article will be made available by the authors, without undue reservation.
