# Supplementary material for: Metformin Alleviates LPS-Induced Acute Lung Injury by Regulating the SIRT1/NF-κB/NLRP3 Pathway and Inhibiting Endothelial Cell Pyroptosis
Source: Front Pharmacol. 2022 Jul 15;13:801337. doi: 10.3389/fphar.2022.801337 (PMC9334876; doi:10.3389/fphar.2022.801337)
Supplement: Supplementary file 1 [file DataSheet1.docx]

Supplemental Digital Content

**Metformin alleviates LPS-induced acute lung injury by regulating the SIRT1/NF-κB /NLRP3 pathway and inhibiting endothelial cell pyroptosis**

Yunqian Zhang^1^*, Hui Zhang^1^*, Siyuan Li^1^*, Kai Huang^1^, Lai Jiang^1^, Yan Wang^1^

1-Department of Anesthesiology and Surgical Intensive Care Unit, Xinhua Hospital affiliated to Shanghai Jiaotong University School of Medicine, Shanghai, 200092, China

Corresponding authors

Dr. Yan Wang

Department of Anesthesiology and Surgical Intensive Care Unit, Xinhua Hospital, Shanghai Jiaotong University School of Medicine, 1665 Kongjiang Road, Shanghai, 200092, China. Email: wangyan@xinhuamed.com.cn

Dr. Lai Jiang

Department of Anesthesiology and Surgical Intensive Care Unit, Xinhua Hospital, Shanghai Jiaotong University School of Medicine, 1665 Kongjiang Road, Shanghai, 200092, China.

Email: jianglai@xinhuamed.com.cn

**Supplemental Figure S1**


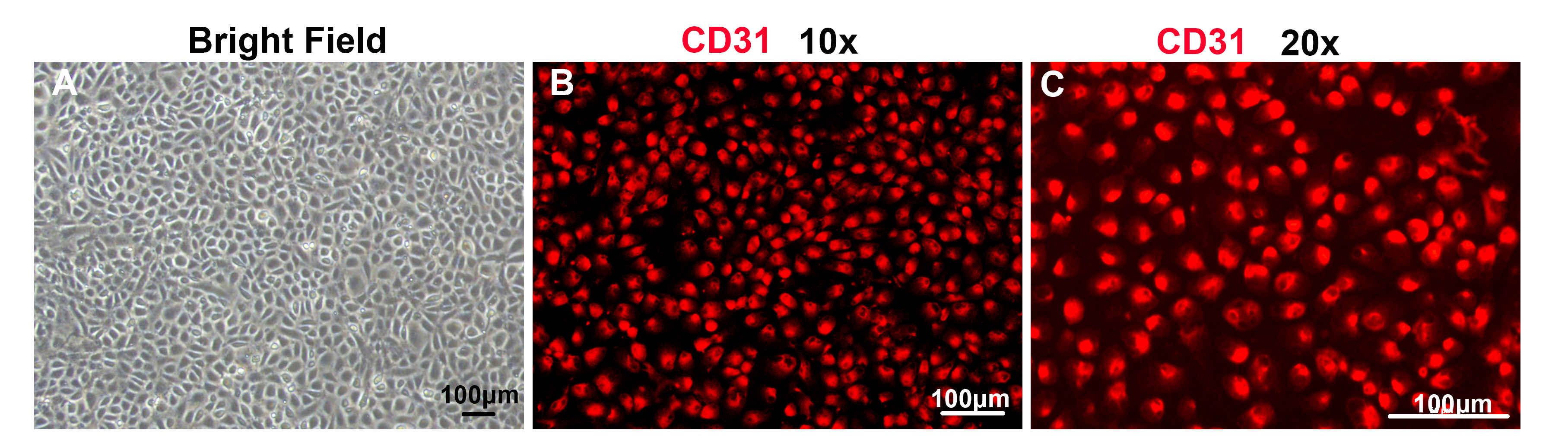


**Supplemental Figure S1. Identification of mouse pulmonary vascular endothelial cells.** ECs were cultured and stained with endothelial marker CD31**.** (A) Morphology of endothelial cells in bright field, Original magnification, × 50. (B) Endothelial cells were stained with CD31, Original magnification, × 100. (C) Endothelial cells were stained with CD31, Original magnification, × 200, Scale bar indicates 100μm.

**Supplemental Figure S2**


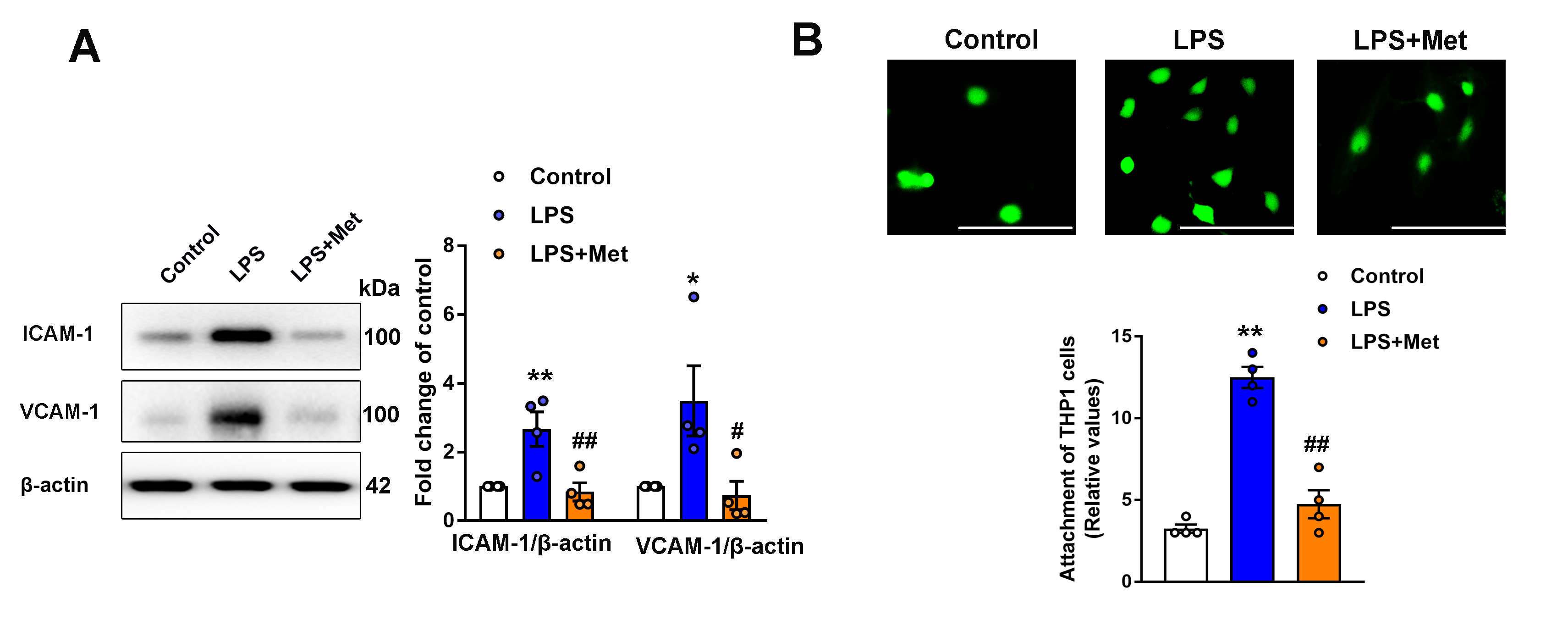


**Supplemental Figure S2. Metformin inhibited LPS-induced expression of endothelial cell adhesion molecules and monocytes-endothelial attachment.** (A) Protein expression of ICAM-1 and VCAM-1 were determined by western blotting. Relative densitometry of the ICAM-1 and VCAM-1 protein band over β-actin were shown in histogram. Representative protein bands were presented on the left of the bar graphs. The monocytes attached to endothelial cells were labeled with Calcein-AM with green fluorescence. (B) Representative images of adhesive THP-1 cells and quantification of adhesive THP-1 cells were shown. Original magnification, × 200. Scale bar indicates 100 μm. Data are expressed as means ± SEM (n = 4). **P*<0.05, ***P*<0.01 vs. Control group; ^#^*P*<0.05, ^##^*P*<0.01 vs. LPS group.

**Supplemental Figure S3**


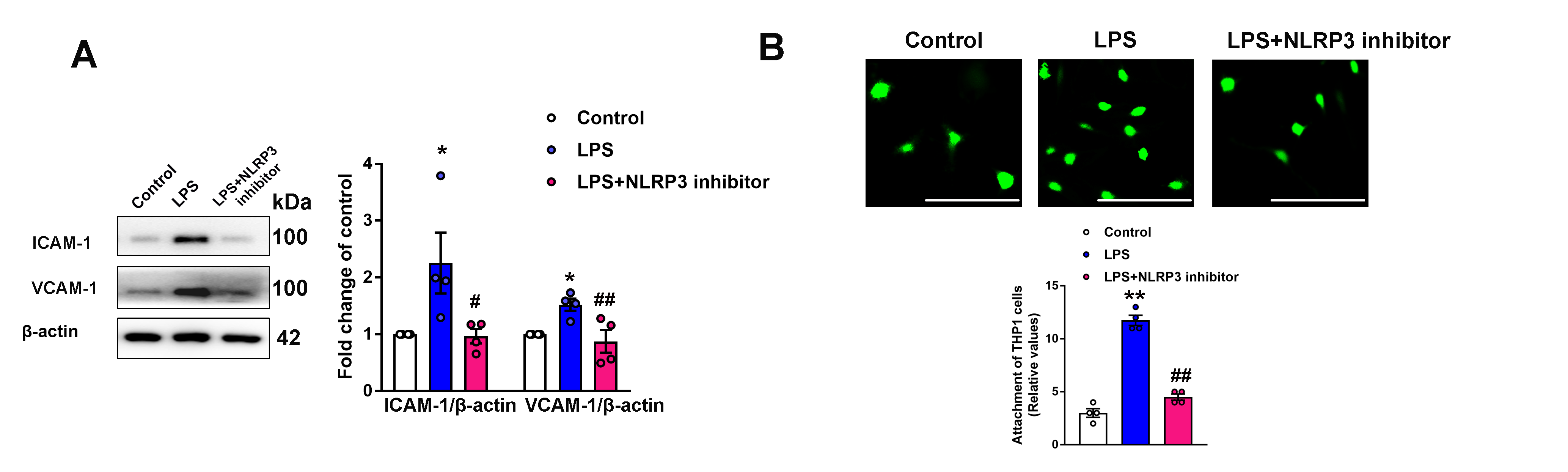


**Supplemental Figure S3. NLRP3 inhibitor MCC950 inhibited LPS-induced expression of endothelial cell adhesion molecules and monocytes-endothelial attachment.** (A) Representative protein bands of ICAM-1 and VCAM-1 were presented on the left of the bar graphs. (B) Representative images of adhesive THP-1 cells and quantification of adhesive THP-1 cells were shown. Original magnification, × 200. Scale bar indicates 100 μm. Data are expressed as means ± SEM (n = 4). **P*<0.05, ***P*<0.01 vs. Control group; ^#^*P*<0.05, ^##^*P*<0.01 vs. LPS group.

**Supplemental Figure S4**


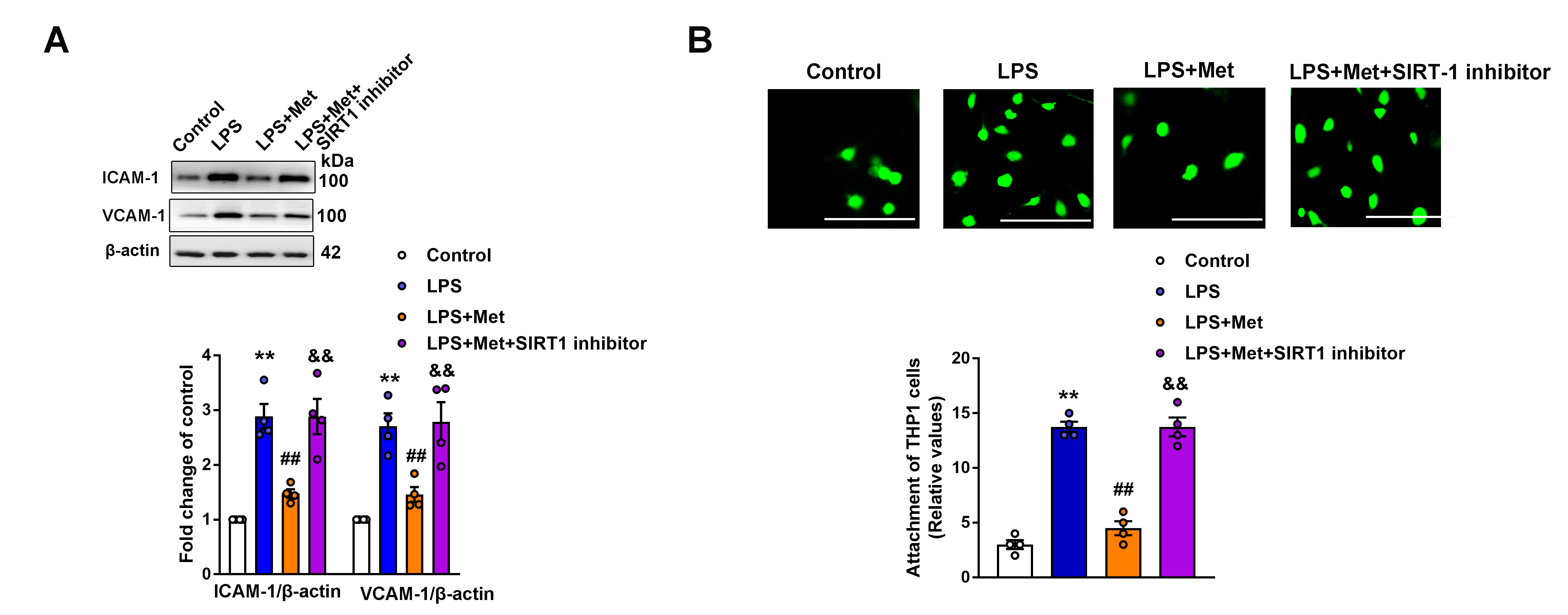


**Supplemental Figure S4. The SIRT1 inhibitor nicotinamide prevents metformin from reducing LPS-induced expression of endothelial cell adhesion molecules and monocytes-endothelial attachment in pulmonary ECs.** (A) Representative protein bands of ICAM-1 and VCAM-1 and relative densitometry were shown. (B) Representative images and quantification of adhesive THP-1 cells were shown. Original magnification, × 200. Scale bar indicates 100 μm. Data are expressed as means ± SEM (n = 4). ***P*<0.01 vs. Control group; ^##^*P*<0.01 vs. LPS group; ^&&^*P*<0.01 vs. LPS+Met group.

**Supplemental Figure S5**


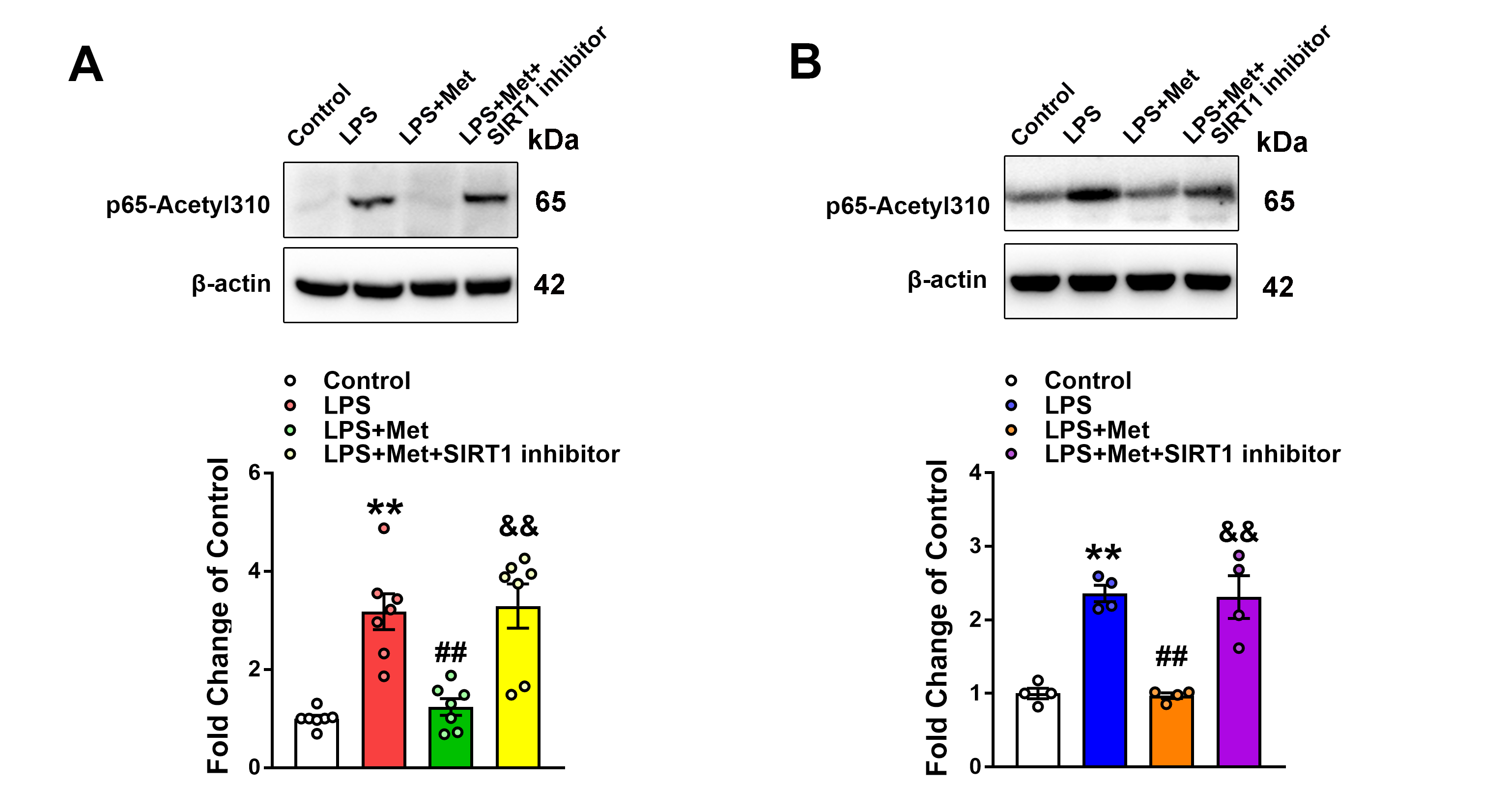


**Supplemental Figure S5. SIRT-1 inhibitor treatment increased the expression of p65 lysine 310 acetylation both *in vivo* and *in vitro*.** (A) Representative p65 lysine 310 acetylation protein bands in mouse lung tissues, relative densitometry were shown in histogram under the bands. Data are expressed as means ± SEM (n = 7). (B) Representative p65 lysine 310 acetylation protein bands in mouse pulmonary ECs, relative densitometry were shown in histogram. Data are expressed as means ± SEM (n = 4). ***P*<0.01 vs. Control group; ^##^*P*<0.01 vs. LPS group; ^&&^*P*<0.01 vs. LPS+Met group.

**Supplemental Figure S6**


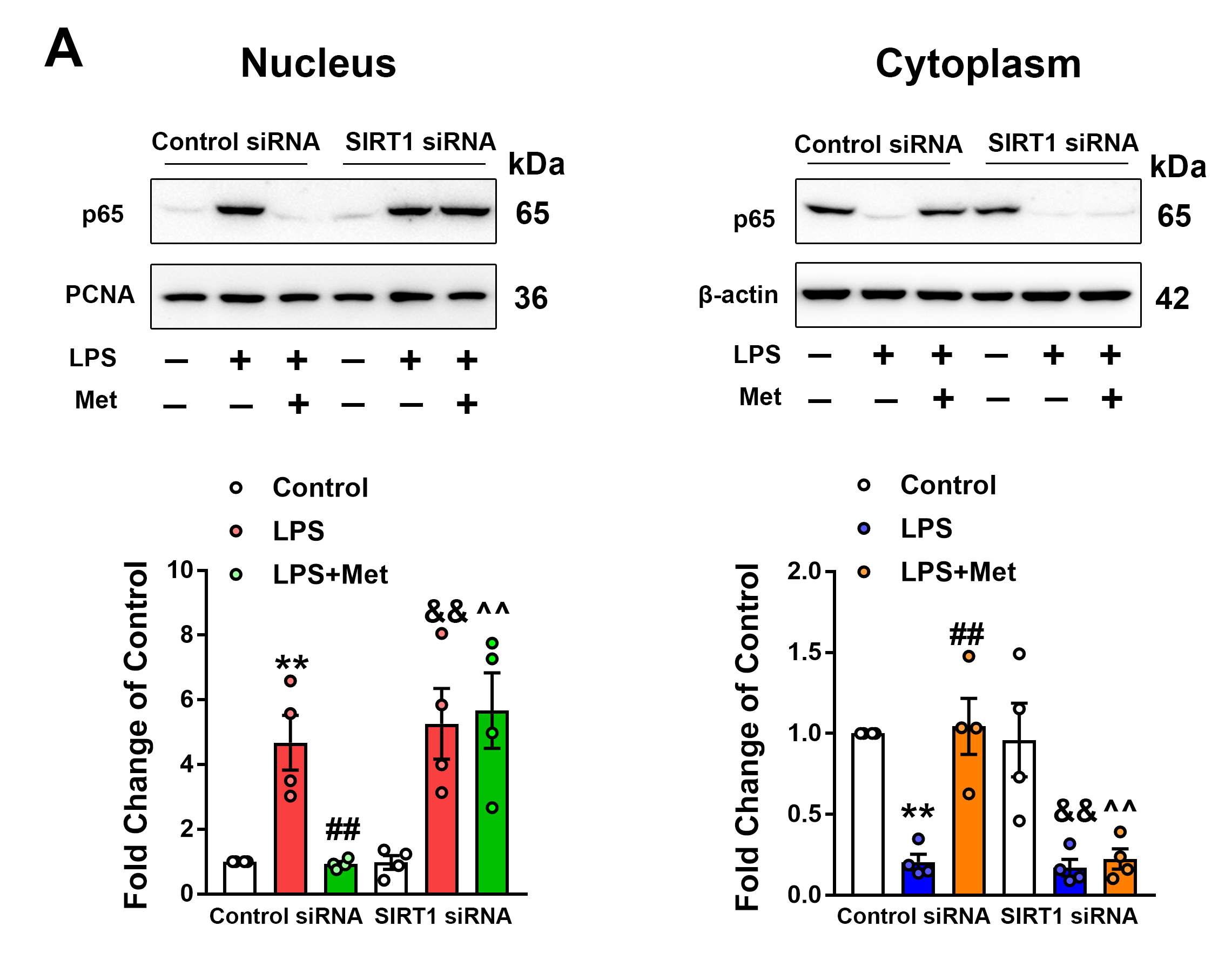


**Supplemental Figure S6. SIRT1 siRNA reversed the effect of metformin on the increased NF-κB signaling pathway induced by LPS.** (A) SIRT1 siRNA and Control siRNA were added to pulmonary ECs 24 h before LPS and metformin treatment. Cells were harvested and protein was extracted using nuclear and cytoplasmic protein extraction Kit. (A) NF-κB p65 protein expression in nucleus was detected, relative densitometry of the p65 protein band over PCNA were shown in histogram. (B) NF-κB p65 protein expression in cytoplasm was detected, relative densitometry of the p65 protein band over β-actin were shown. Data are expressed as means ± SEM (n = 4). ***P*<0.01 vs. Control siRNA group; ^##^*P*<0.01 vs. Control siRNA +LPS group; ^&&^*P*<0.01 vs. SIRT1 siRNA group. ^^*P*<0.01vs. Control siRNA +LPS+Met group.
